# Bioassay-guided isolation and identification of gametocytocidal compounds from *Artemisia afra* (Asteraceae)

**DOI:** 10.1186/s12936-019-2694-1

**Published:** 2019-03-08

**Authors:** Phanankosi Moyo, Phaladi Kunyane, Mamoalosi A. Selepe, Jacobus N. Eloff, Jandeli Niemand, Abraham I. Louw, Vinesh J. Maharaj, Lyn-Marie Birkholtz

**Affiliations:** 1 Malaria Parasite Molecular Laboratory, Department of Biochemistry, Genetics and Microbiology, Faculty of Natural and Agricultural Sciences, Institute for Sustainable Malaria Control, University of Pretoria, Private Bag x20, Hatfield, 0028 South Africa; 2 Department of Chemistry, Faculty of Natural and Agricultural Sciences, Institute for Sustainable Malaria Control, University of Pretoria, Private Bag x20, Hatfield, 0028 South Africa; 3 Phytomedicine Programme, Department of Paraclinical Sciences, Faculty of Veterinary Science, University of Pretoria, Private Bag x04, Onderstepoort, Pretoria, 0110 South Africa

**Keywords:** Malaria, Gametocytes, Transmission-blocking, *Artemisia afra*, Sesquiterpene lactone, Natural products, *Plasmodium falciparum*

## Abstract

**Background:**

Optimal adoption of the malaria transmission-blocking strategy is currently limited by lack of safe and efficacious drugs. This has sparked the exploration of different sources of drugs in search of transmission-blocking agents. While plant species have been extensively investigated in search of malaria chemotherapeutic agents, comparatively less effort has been channelled towards exploring them in search of transmission-blocking drugs. *Artemisia afra* (Asteraceae), a prominent feature of South African folk medicine, is used for the treatment of a number of diseases, including malaria. In search of transmission-blocking compounds aimed against *Plasmodium* parasites, the current study endeavoured to isolate and identify gametocytocidal compounds from *A. afra*.

**Methods:**

A bioassay-guided isolation approach was adopted wherein a combination of solvent–solvent partitioning and gravity column chromatography was used. Collected fractions were continuously screened in vitro for their ability to inhibit the viability of primarily late-stage gametocytes of *Plasmodium falciparum* (NF54 strain), using a parasite lactate dehydrogenase assay. Chemical structures of isolated compounds were elucidated using UPLC-MS/MS and NMR data analysis.

**Results:**

Two guaianolide sesquiterpene lactones, 1α,4α-dihydroxybishopsolicepolide and yomogiartemin, were isolated and shown to be active (IC_50_ < 10 μg/ml; ~ 10 μM) against both gametocytes and intra-erythrocytic asexual *P. falciparum* parasites. Interestingly, 1α,4α-dihydroxybishopsolicepolide was significantly more potent against late-stage gametocytes than to early-stage gametocytes and intra-erythrocytic asexual *P. falciparum* parasites. Additionally, both isolated compounds were not overly cytotoxic against HepG2 cells in vitro.

**Conclusion:**

This study provides the first instance of isolated compounds from *A. afra* against *P. falciparum* gametocytes as a starting point for further investigations on more plant species in search of transmission-blocking compounds.

**Electronic supplementary material:**

The online version of this article (10.1186/s12936-019-2694-1) contains supplementary material, which is available to authorized users.

## Background

With over 1000 genera, 13 tribes and 20,000 species, the Asteraceae is one of the most diverse and largest flowering plant families in the world [1]. In addition to its economic and ornamental value, this plant family is well renowned for its medicinal prowess and commonly features in folk medicine [2]. It is widely adopted in traditional medicinal practice for the treatment of a large number of ailments ranging from wound healing and coughs to fever [3]. The Asteraceae family has similarly made an invaluable mark within the mainstream medicinal sector, particularly in the malaria field, with the discovery of the highly potent and fast-acting antiplasmodial sesquiterpene lactone compound, artemisinin, from the Chinese medicinal herbal species *Artemisia annua* (Asteraceae) [4]. Artemisinin derivatives play a pivotal role in malaria control where they are used as main partner drugs in artemisinin-based combination therapy (ACT), which currently serve as first-line treatment drugs for malaria [5]. ACT primarily acts on the symptomatic intra-erythrocytic asexual *Plasmodium falciparum* parasites but, unfortunately, most have limited transmission-blocking activity [6].

With the shift in global malaria management programmes from control to eradication of the disease, the transmissible, asymptomatic, intra-erythrocytic sexual gametocyte stages of *Plasmodium* parasites have been identified as a priority target for transmission-blocking efforts [7]. However, optimal adoption of this strategy is currently hindered by lack of safe and effective transmission-blocking drugs [7]. While ACT reduces gametocyte carriage, it does not have a sterilizing effect on mature stage-V transmissible gametocytes and consequently does not reduce transmission of *Plasmodium* parasites [8, 9]. The use of primaquine, the only World Health Organization-recommended malaria transmission-blocking drug, is severely restricted by its toxicity [10]. This limited armoury of transmission-blocking drugs has necessitated exploration of different sources of drugs in search of novel compounds that are potent against gametocyte stages of *Plasmodium* parasites [11–16]. One potential vast source that still remains relatively unscrutinized in this effort is medicinal plant species [17].

Despite the limited interrogation of plant species in search of transmission-blocking compounds, crude extracts of plants and plant-derived compounds have encouragingly been shown to be potent against late-stage gametocytes of *Plasmodium* parasites, both in vitro and in vivo [17–21]. Asteraceae is emerging as a promising potential candidate source worth interrogating in the search for transmission-blocking drugs, primarily because these plants produce high levels of sesquiterpene lactones with good gametocytocidal activities [17, 18, 22]. Crude water extract of *Vernonia amygdalina* (Asteraceae) has in vivo activity against *Plasmodium berghei* gametocytes with pronounced potency on microgametocytes compared to macrogametocytes [18]. Two sesquiterpene lactones, parthenin and parthenolide, derived from *Parthenium hysterophorus* (Asteraceae) and *Tanacetum parthenium* (Asteraceae), respectively, are active against mature stage-V gametocytes of *P. falciparum* [22]. Based upon this demonstrated gametocytocidal activity, there is merit in further examination of other medicinal plant species within the Asteraceae family in search of *Plasmodium* transmission-blocking drugs, and one species which warrants exploration is *Artemisia afra* (Asteraceae) [17].

*Artemisia afra* is a commonly used indigenous medicinal plant species in South Africa for the treatment of colds, coughs and fever [3, 23]. Its use in the treatment of fever led to studies on its antiplasmodial properties in search of novel chemotherapeutic anti-malarial agents [24–26]. In search of a novel transmission-blocking lead, the in vitro potency of *A. afra* crude extract against the transmissible late-stage gametocytes of chloroquine-sensitive *P. falciparum* parasites was reported. However, the identity of the gametocytocidal compounds in *A. afra* was not determined [17]. The present study provides a report on the bioassay-guided isolation and identification of two active compounds associated with potent gametocytocidal activity of *A. afra.* The agents, a recent study investigated and provided fractionation steps for isolation procedure were primarily guided by in vitro activity against late-stage gametocytes of *P. falciparum* as measured using the parasite lactate dehydrogenase (pLDH) assay [17, 27].

## Methods

### Plant material collection, drying, extraction, and solvent–solvent partitioning of crude extract

Leaves of *A. afra* were collected from the Manie van der Schijff Botanical Garden at the University of Pretoria in July 2015. *Artemisia afra* leaves were collected from the same plant used for an earlier study [17]. Voucher specimens were previously collected and deposited at the H. G. W. J. Schweickerdt Herbarium of the University of Pretoria (Voucher specimen number PRU 121 389) [17]. Plant material was dried, ground to fine powder and extracted using acetone as previously described [17]. Dried crude extract of *A. afra* was subjected to solvent–solvent partitioning using five solvent systems of varying polarity in sequential order. The crude extract was dissolved in distilled water and extracted three times with hexane. After separation, the water was extracted separately with chloroform, ethyl acetate and methanol. The organic solvents were kept separate and evaporated to dryness under a stream of air.

### In vitro *Plasmodium falciparum* NF54 culturing, viability and cytotoxicity assays

The chloroquine-sensitive *P. falciparum* (NF54 strain) was cultured as described by Verlinden et al. [28] and homogenous ring-stage intra-erythrocytic asexual *Plasmodium* parasites generated through d-sorbitol (5% w/v) synchronization. Gametocytogenesis initiation, induction and gametocyte culturing was done as described by Reader et al. [29].

The effect of dried fractions of *A. afra* and isolated compounds (10 mg/ml in 100% DMSO; highest DMSO concentration of < 0.4% in assays) on the viability of both intra-erythrocytic asexual parasites and gametocytes stages of *P. falciparum* were assessed using the pLDH assay [17, 27] with slight modifications for asexual assays where a young trophozoite culture of *P. falciparum* (1% parasitaemia) was incubated for 72 h after which pLDH activity was quantified spectrophotometrically [29]. Both methylene blue (MB) and artemisinin (ART) was used as gametocytocidal drug controls and chloroquine for asexual drug control.

The LDH leakage assay was used to examine the in vitro cytotoxicity of the dried chloroform fraction of *A. afra* as well as that of the active compounds isolated (at concentrations of 2.5, 5, 10, and 20 µg/ml) in the study. Cytotoxicity assays were assessed on the HepG2 cell line (20,000 cells/well) as previously described [28]. Emetine was as used as cytotoxic positive control.

### Statistical analysis

Assay data were normalized against untreated *Plasmodium* parasites and HepG2 cell controls. Full dose–response assays (twofold serial dilution) on *Plasmodium* parasites were carried out over a concentration range of 0.0003–40 μg/ml. Data were analysed on Microsoft Excel and sigmoidal dose–response curves were plotted using GraphPad Prism (v5). Unless otherwise stated, all data have been expressed as mean ± SEM (*n *≥ 2, carried out in three technical repeats). Statistical analysis was carried out on GraphPad Prism (v5) using unpaired Student-*t* test with a level of significance of *P *< 0.05.

### Bioassay-guided fractionation of *Artemisia afra* chloroform fraction, isolation and purification of gametocytocidal compounds

To isolate and purify gametocytocidal compounds from *A. afra*, gravity column chromatography was used. The dried chloroform fraction produced from the solvent–solvent partitioning step (and shown in vitro using the pLDH assay to be the most potent against late-stage gametocytes) was fractionated by primary column chromatography (designated column 1, eluent of hexane and ethyl acetate with increasing polarity as follows: hexane: ethyl acetate—7:3–1:1–4:6). The collected fractions from column 1 (F1–F24) were screened in vitro against late-stage gametocytes of *P. falciparum* using the pLDH assay, following which fractions F13 and F19 were subsequently prioritized for further purification. Fraction F13 was purified using secondary and tertiary columns (designated columns 2 and 3 with eluents of hexane: dichloromethane: acetone at ratios of 5:5:4 and 1:4:1, respectively), while F19 was purified using secondary column chromatography (designated column 4 with eluent of hexane: dichloromethane: acetone at ratio of 5:5:3). For column 1, all solvents used were technical grade, while for columns 2, 3 and 4 only analytical grade solvents from Merck were used. All column chromatography fractionation steps were carried out using silica gel 60 (0.063–0.2 mm particle size, Fluka Chemie, Buchs, Switzerland).

To monitor separation of compounds during fractionation, thin layer chromatography (TLC) [30] and ultra-high pressure liquid chromatography coupled to a mass spectrometer (UPLC-MS) phytochemical profiling were employed. For UPLC-MS analysis, samples were analysed using a Waters Acquity™ UPLC instrument coupled to a Waters Synapt G2 high definition MS. All samples were prepared at 1 mg/ml using the same binary solvent system (water and acetonitrile—1:1 v/v) with an injection volume of 5 µl per run. Samples were run on an Acquity UPLC™ BEH C_18_ column (1.7 µm particle size, 100 mm × 2.1 mm, Waters, Ireland) using 0.1% (v/v) formic acid in water (eluent A) (Fluka Analytical, Sigma-Aldrich, Switzerland) and 0.1% (v/v) formic acid in acetonitrile (eluent B) (Ultra gradient, ROMIL Ltd, Cambridge, UK). Solvent gradient varied over time as follows; eluent A%: eluent B%—time; 97: 3–0.1 min; 80: 20–6 min; 70: 30–9 min; 70: 30–9.2 min; 0: 100–20 min; 0: 100–23.5 min; 97: 3–24 min; 97: 3–25 min. Flow rate was set at 0.4 ml/min at a pressure of 0–15,000 psi. Mass spectral data were acquired using electrospray ionization in positive mode (ESI +ve mode). The conditions were set as follows: 2.6 kV capillary voltage, 30 V sampling voltage, 4 V extraction cone voltage, 120 °C source temperature and 600 °C desolvation temperature. Scan mass range was set for 50–1200 m/z. MassLynx (Version 4.6) software was used for data acquisition and processing.

### NMR analysis

1D (^1^H, ^13^C and ^13^C DEPT-135) and 2D (HSQC, HMBC, NOESY and COSY) NMR experimental spectra were recorded at room temperature, on a Bruker Avance III 400 spectrometer, operating at 400 MHz for ^1^H NMR and 100 MHz for ^13^C NMR. The isolated compounds from *A. afra* were dissolved in deuterated chloroform (CDCl_3_) (Aldrich Chemistry, Sigma-Aldrich, USA). The chemical shifts from ^1^H NMR and ^13^C NMR spectra are reported in parts per million relative to the solvent residual peak (δ_H_—7.26 and δ_C_—77.0).

## Results

### Bioassay-guided fractionation and isolation of gametocytocidal compounds from *Artemisia afra*

**Fig. 1:**
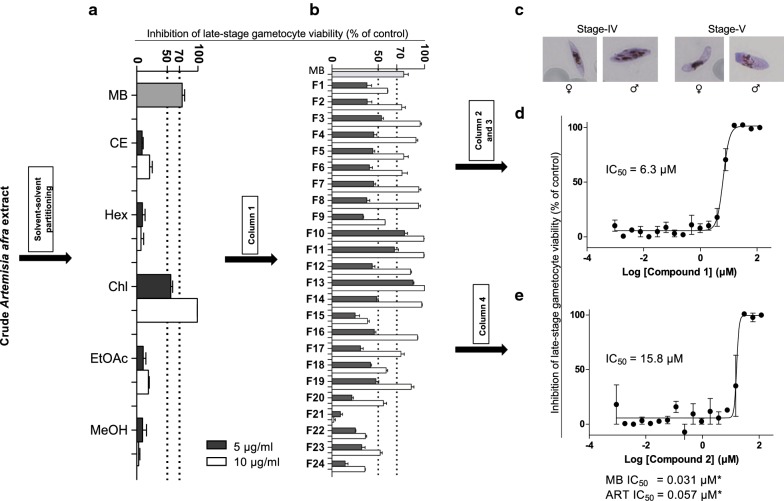
Bioassay-guided fractionation and isolation of gametocytocidal compounds 1 and 2 from *Artemisia afra.* Inhibition of in vitro viability of late-stage gametocytes (1.2–1.6% gametocytaemia, *n *= 1) of *P. falciparum* (NF54 strain) by crude extract and fractions of *Artemisia afra*, realised from **a** solvent–solvent partitioning step and **b** separation of chloroform fraction (column 1). **c** Typical microscopic pictures of macrogametocytes (condensed nuclei) and microgametocytes (scattered nuclei) used in pLDH assays. Full dose–response investigation of compound **d 1** and **e 2** against late stage gametocytes (1–2% gametocytaemia) of *P. falciparum*. IC_50_ values (μM), determined using the 72 + 72 h pLDH assay. Data are the mean ± SEM of three independent biological repeats in technical triplicates. Positive drug control for inhibition of viability of late-stage gametocytes were methylene blue and artemisinin. *Unpaired assays (*n *= 1, each carried out in technical triplicate, see Additional file 1: Fig. S2). *MB* methylene blue, *Hex* hexane, *CE* crude extract, *Chl* chloroform, *EtOAc* ethyl acetate, *MeOH* methanol, *ART* artemisinin

From an initial 400 g of dried *A. afra* leaves, 21.5 g of crude acetone extract was obtained. Subsequent solvent–solvent partitioning of crude extract (21.1 g) yielded four fractions, namely hexane (9.2 g), chloroform (8.2 g), ethyl acetate (1.4 g), and methanol (2.2 g) (Fig. 1a). Following the solvent–solvent partitioning procedure the next step was to identify the most potent fraction, as observed by inhibition of in vitro *P. falciparum* late-stage gametocyte viability (> 85% stage IV–V).

The activity of crude extract of *A. afra* and the four dried fractions (at dual point concentrations of 5 and 10 µg/ml) on the viability of late stage gametocytes of *P. falciparum* was assessed using the pLDH assay (Fig. 1a, see Additional file 1: Table S1, *n *= 1). Methylene blue (10 μM) served as positive control drug inhibiting > 70% in vitro viability of late gametocyte stages. The hexane and methanol fractions were the least active (< 10% inhibition). By contrast, the chloroform fraction was potently active against late-stage gametocytes, inhibiting viability of these *P. falciparum* parasites at 56–99%, compared to 9–21% inhibition seen with the crude extract (Fig. 1a). Further purification of the chloroform fraction with solvent–solvent partitioning (column 1) resulted in 24 fractions, F1–F24 (Fig. 1b). From these, 15 fractions where highly active (> 70% inhibition of gametocyte viability at 10 µg/ml) against late stage gametocytes of *P. falciparum* (Fig. 1b; see Additional file 1: Table S2). Fractions F10, F11 and F13 were highly potent and retained > 70% inhibition of in vitro late-stage gametocyte viability even at 5 µg/ml. A subsequent stringent three-point selection criterion was employed to select fractions for additional column chromatography purification: (i) bio-activity (≥ 50% and > 85% inhibition of in vitro gametocyte viability at 5 and 10 μg/ml, respectively); (ii) mass of collected fraction (> 0.3 g); and (iii) purity as assessed from UPLC-MS data analysis (see Additional file 1: Fig. S1). Only two fractions, F13 and F19, passed this criterion and were subjected to gravitational column chromatography to isolate the gametocytocidal compounds therein.

Secondary column chromatography purification (column 2) of F13 yielded a crystalline compound of colourless to off-brown colour on visual inspection as the major compound in this fraction. The presence of an off-brown colour indicated presence of an impurity in the major compound, which necessitated a final column chromatography purification step (column 3) yielding clear ‘needle-like’ crystals, designated compound **1**. Secondary column chromatography (column 4) purification of fraction F19 resulted in isolation of a white powder designated compound **2**.

In vitro full dose–response activity of compounds **1** and **2** against late-stage gametocytes (Fig. 1c) of *P. falciparum* (Fig. 1d, e) indicated that both compounds had good activity (criteria for ‘good’ activity, IC_50_ < 10 µg/ml, as defined by Batista et al. [31] has been adopted in this current study). Compound **1** was significantly more active against late-stage gametocytes (IC_50_ = 6.3 µM (2 µg/ml)) compared to compound **2** (IC_50_ = 15.8 µM (5.3 µg/ml)) (Fig. 1d, e, *n *= 3, *P *< 0.05, unpaired Student’s *t* test).

**Fig. 2:**
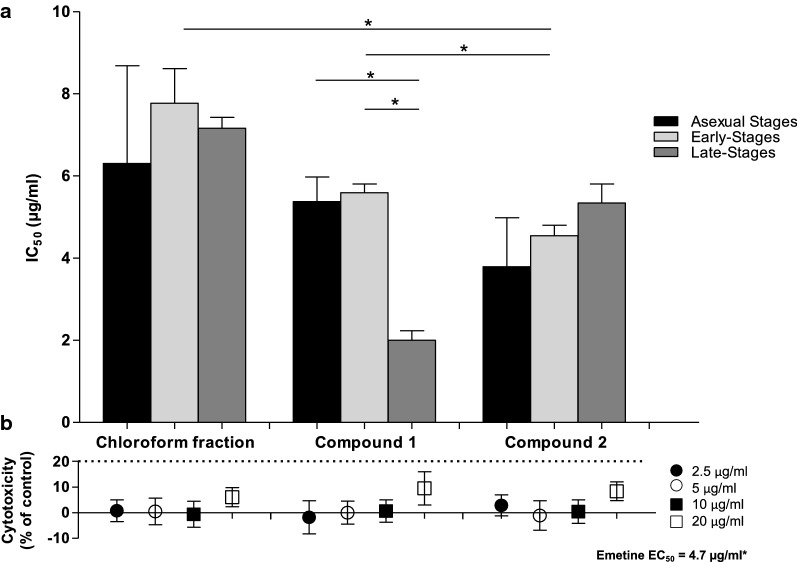
Pan-reactivity and cytotoxic investigations of *Artemisia afra* chloroform fraction, compounds 1 and 2. **a** IC_50_ values (μM), determined using the pLDH assay for asexuals (1% parasitaemia) and gametocytes (1–2% gametocytaemia), respectively. Data are the mean ± SEM of three independent biological repeats each done in technical triplicate. *n *= 2 for all asexual assays. Statistically significant differences between the IC_50_ values are indicated (*P < 0.05, unpaired Student’s *t*-test). **b** Cytotoxicity was investigated by the standard mammalian LDH leakage assay, with emetine used as positive reference control. *Unpaired assays (*n *= 3, each carried out in technical duplicate). Data are the mean ± SEM of three independent biological repeat experiments each performed in technical triplicate

Motivated by the need for discovery of pan-reactive anti-malarial drugs, compounds **1** and **2** were additionally screened in vitro against early-stage gametocytes (> 85% stage II–III) and intra-erythrocytic asexual *P. falciparum* parasites using the pLDH assay (Fig. 2a; see Additional file 1: Table S3). The chloroform fraction was screened parallel to both compounds **1** and **2**. The chloroform fraction, compounds **1** and **2** showed good activity across all three intra-erythrocytic parasite stages (Fig. 2a). Although there was no statistically significant (*n *= 2, *P *> 0.05, unpaired Student’s *t*-test) difference between the activities of compounds **1** and **2** and that of the parent chloroform fraction of *A. afra* against asexual parasites of *Plasmodium*, compound **1** was significantly (*n *≥ 2, *P *< 0.05, unpaired Student’s *t*-test) more potent against late-stage gametocytes compared to both early-stage gametocytes and intra-erythrocytic asexual parasites of *P. falciparum*. By contrast, compound **2** was the most potent against early-stage gametocytes compared to compound **1** and the chloroform fraction (*n *= 3, *P *< 0.05, unpaired Student’s *t*-test).

To exclude the possibility of cellular toxicity as a cause for the observed pan-reactivity, in vitro cytotoxicity of compounds **1** and **2** was interrogated using the LDH leakage assay against HepG2 cells and compared to that of the parent chloroform fraction of *A. afra* (Fig. 2b). Emetine (100 µM) was used as positive control drug giving > 50% cytotoxicity with a measured EC_50_ value of 4.7 µg/ml. The chloroform fraction and compounds **1** and **2** showed minimal cytotoxicity with < 10% toxicity seen at 20 µg/ml (Fig. 2b). In vitro cytotoxicity of compounds **1** and **2** ranged from 0.03–9.47% and 0.40–8.35%, respectively.

### Structure elucidation of isolated compounds

The structures and identity of compounds **1** and **2** was subsequently elucidated. Compound **1** was isolated as colourless ‘needle-like’ crystals. The HR-ESI–MS of **1** gave an [M+Na]^+^ ion peak at *m/z* 343.1167 suggesting a molecular formula of C_17_H_20_O_6_ (calcd [M+Na]^+^ *m/z* 343.1158). The NMR assignments for the compound are shown in Table 1. All proton and carbon signals were assigned on the basis of 1D (^1^H, ^13^C and DEPT-135) and 2D (HSQC, 1H–1H COSY, and HMBC) NMR experiments. The ^1^H NMR spectrum showed signals for two pairs of exomethylene protons at δ_H_ 5.13 (1H, brs, H-14a), 4.86 (1H, brs, H-14b), 6.32 (1H, d, *J* = 3.4, H-13a) and 5.86 (1H, d, *J* = 3.4, H-13b), while signals for the 2,3 alkene protons resonated at δ_H_ 5.62 (1H, d, *J* = 5.6, H-2) and 6.00 (1H, d, *J* = 5.6, H-3). Two singlets integrating for three protons each were observed at δ_H_ 2.14 and 1.32 for the acetyl methyl and vinyl methyl signals, respectively. Additionally, four methine signals appeared at δ_H_ 2.51 (1H, d, *J* = 11.4, H-5), 4.16 (1H, dd, 8.5, *J* = 11.4, H-6), 3.63–3.70 (1H, m, H-7) and 4.86–4.93 (1H, m, H-8), as well as the signals for the methylene protons at δ_H_ 2.90 (1H, dd, *J* = 6.0, 12.0, H-9a) and 2.73 (1H, dd, *J* = 10.5, 12.0, H-9b). The ^13^C NMR showed the presence of 17 carbon atoms with two signals at δ_C_ 169.9 and at δ_C_ 169.8 indicating the presence of two carbonyl groups. The signals appearing at δ_C_ 142.9, 140.6, 135.5, 134.4, 125.3, and 117.4 pointed to the presence of the six olefinic carbon atoms. The DEPT-135 showed the presence of two methyl, three methylene, six methine and six quaternary carbon atoms.

**Table 1:** ^13^C and ^1^H NMR spectroscopic data for compounds 1 and 2 in CDCl_3_

Po*s*ition	Compound 1		Compound 2	
	δ_**C**_	δ_H_ (*J* in Hz)	δ_C_	δ_H_ (*J* in Hz)
1	85.4	–	76.5	–
2	134.4	5.62 (1H, d, 5.6)	58.5	3.67 (1H, brs^b^)
3	140.6	6.00 (1H, d, 5.6)	57.0	3.27 (1H, brs^b^)
4	82.3	–	70.0	–
5	66.3	2.51 (1H, d, 11.4)	44.6	2.85 (1H, d, 10.8)
6	78.1	4.16 (1H, dd, 8.5, 11.4)	76.5	4.24 (1H, t, 10.0)
7	45.0	3.63–3.70 (1H, m)	48.9	3.67–3.77 (1H, m)
8	74.5	4.86–4.93 (1H, m)	71.5^a^	5.25 (1H, m)
9	36.4	2.90 (1H, dd, 6.0, 12.0) 2.73 (1H, dd, 10.5, 12.0)	44.0	2.41 (1H, dd, 7.1, 16.5) 1.77 (1H, dd, 1.7, 16.5)
10	143.0	–	71.2^a^	–
11	135.5	–	137.4	–
12	169.7^a^	–	169.3	–
13	125.3	6.32 (1H, d, 3.4) 5.86 (1H, d, 3.4)	121.5	6.21 (1H, d, 3.1) 5.56 (1H, d, 3.1)
14	117.4	5.13 (1H, brs) 4.86 (1H, brs)	26.6	1.10 (3H, s)
15	24.2	1.32 (3H, s)	20.2	1.56 (3H, s)
–OAc (CH_3_)	21.1	2.14 (3H, s)	21.3	2.13 (3H, s)
–OAc (–C=O)	169.9^a^	–	170.0	–

^a^Signals can be interchanged

^b^Signals appear as broad singlets due to poor resolution

**Fig. 3:**
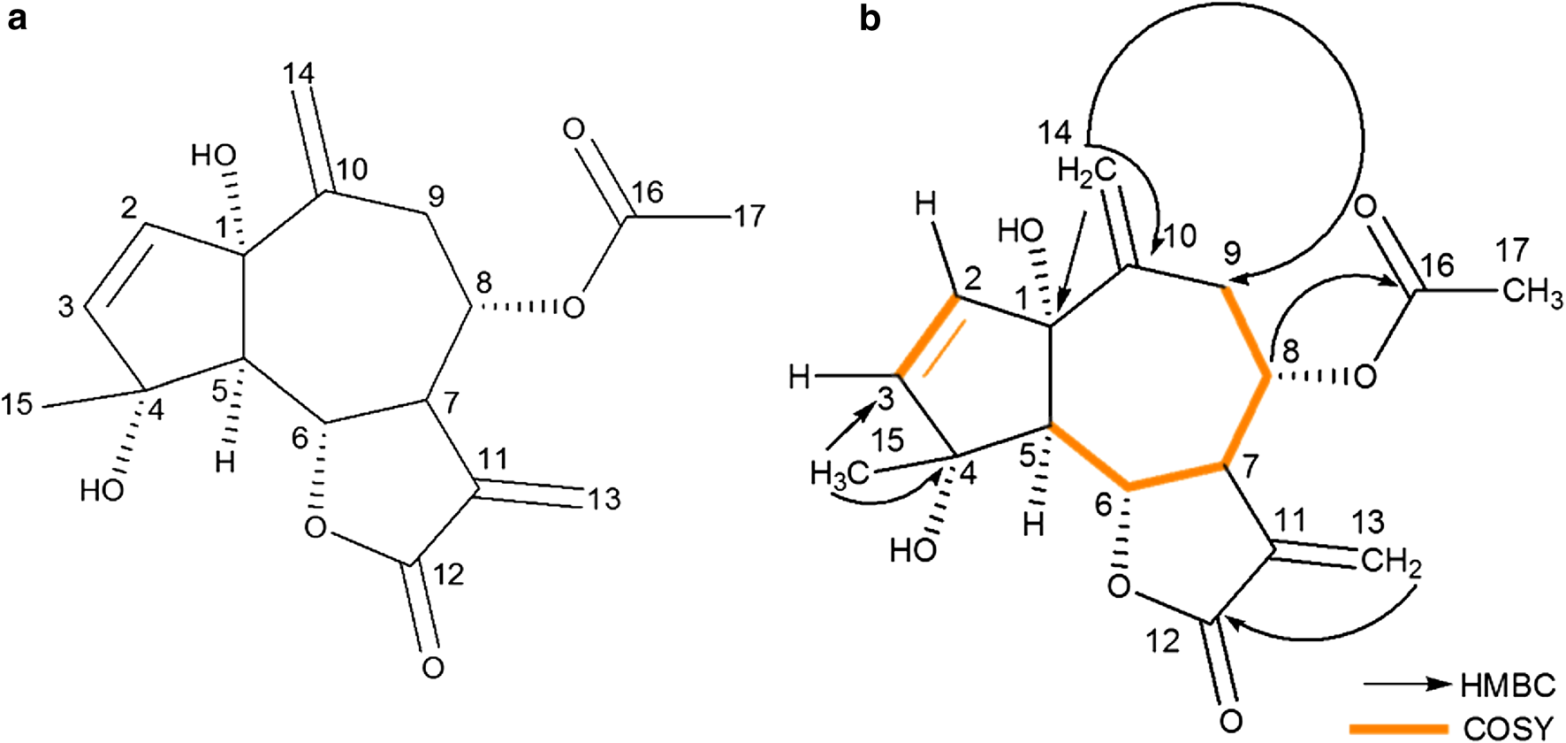
Chemical structure of **a** compound 1, 1α,4α-dihydroxybishopsolicepolide and **b** selected HMBC and COSY correlations

Selected HMBC and COSY correlations for compound **1** are shown Fig. 3. A three-bond HMBC correlation was observed between the exocyclic CH_2_ (H-13a and H13b) and C-12 confirming that the lactone moiety contains the classical exocyclic double bond seen in sesquiterpene lactones. A three-bond correlation between the acetate carbonyl and the proton at C-8 also confirmed that the acetate group is located at position C-8 (Fig. 3). The terminal alkene protons on C14 showed long-range correlation to C-9, and to the quaternary carbon at C-1, thus confirming the position of the terminal double bond in the seven-membered ring. Relative configuration was assigned on the basis of 2D NOESY NMR data. The NMR data for compound **1** matched that previously reported by Singh et al. [32], therefore compound **1** was identified as 1α,4α-dihydroxybishopsolicepolide.

Compound **2** was isolated as a white powder and assigned molecular formula C_17_H_20_O_7_ from its HR-ESI–MS at *m/z* 359.1097 (calcd for [M+Na]^+^ *m/z* 359.1107). The NMR assignments for compound **2** are shown in Table 1. The ^1^H NMR spectrum exhibited doublets at δ_H_ 6.21 (1H, d, *J *= 3.1, H-13a) and δ_H_ 5.56 (1H, d, *J* = 3.1, H-13b), which are characteristic of the exomethylene group. The three singlets upfield resonating at δ_H_ 1.10, 1.56 and 2.13, each integrating for three protons, are assigned to two methyl carbinol groups and one acetyl methyl group. The ^1^H NMR also revealed the presence of four oximethine protons appearing at δ_H_ 3.67 (1H, brs, H-2), 3.27 (1H, brs, H-3), 4.24 9 (1H, t, *J* = 10.0, H-6), and 5.25 (1H, m, H-8), two methine protons resonating at δ_H_ 2.85 (1H, d, *J* = 10.8, H-5) and 3.67–3.77 (1H, m, H-7), and one methylene group appearing at δ_H_ 2.41 (1H, dd, *J* = 7.1 and 16.5 Hz, H-9a) and 1.77 (1H, dd, *J* = 1.7 and 16.5, H-9b). The ^13^C NMR spectrum of compound **2** showed the presence of 17 carbon atoms, which indicated that the compound might be a sesquiterpene. The two signals appearing at δ_C_ 170.0 and 169.3 indicated the presence of two ester-functionalized carbonyl groups for the acetoxy and lactone, respectively. The carbon signals resonating at δ_C_ 137.4 and 121.5 indicated that the alkene is present in the structure. The DEPT-135 spectrum showed the presence of three CH_3_, two CH_2_, six CHs of which four are oxygen linked and six quaternary carbon atoms.

**Fig. 4:**
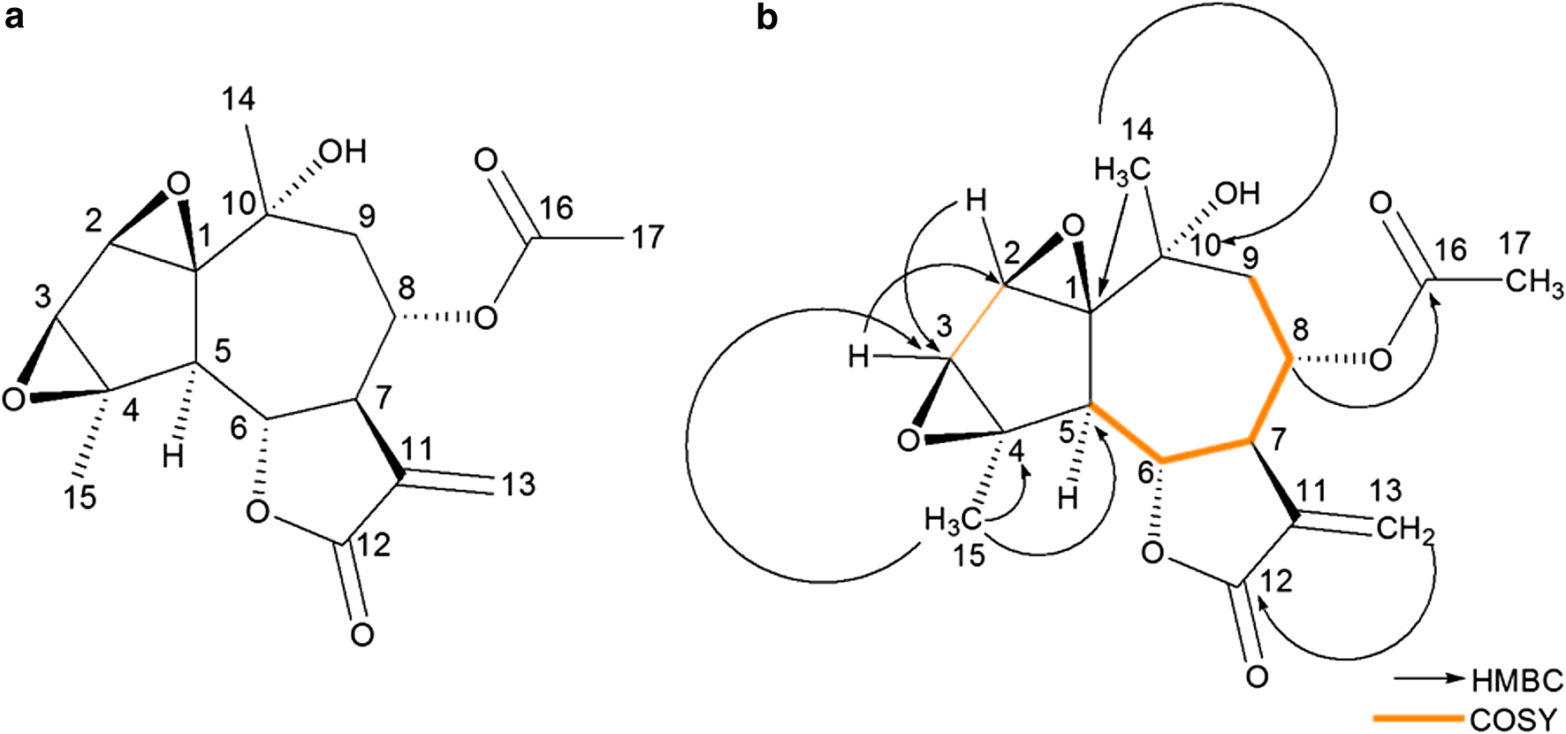
Chemical structure of **a** compound 2, yomogiartemin and **b** selected HMBC and COSY correlations

The key HMBC and COSY connectivity’s of compound **2** are shown in Fig. 4. The position of the lactone functional group was deduced through a three-bond correlation in HMBC spectrum between δ_H_ 6.21 (H-13a), 5.56 (H-13b) and δ_C_ 170.0. A three-bond correlation between oximethine proton appearing at δ_H_ 5.25 (H-8) and the carbonyl of the acetate group showing at δ_C_ 169.3 in the HMBC spectrum confirmed that the acetate group is located at position C-8 of the molecule (Fig. 4). The three-bond HMBC correlation from the protons appearing at δ_H_ 1.10 (3H, s, H-14) to C-1 and C-9 was observed, which suggests that this methyl group is situated at the carbinol carbon C-10. The relationship between H-2 and H-3 was established by the three-bond COSY correlation, which indicated that these protons are adjacent to each other (Fig. 4). Relative configuration was assigned based on 2D NOESY NMR data. Thus, from the NMR data it can be concluded that compound **2** is structurally similar to compound **1**, except that the 2, 3 and 10, 14 double bonds are oxidized to an epoxide and an alcohol, respectively.

The presence of epoxide moieties was evidenced by downfield shifted proton signals resonating at δ_H_ 3.67 (H-2) and 3.27 (H-3) on the ^1^H NMR; these chemical shift signals are consistent to those reported for epoxide protons found on a cyclopentane ring of a guaianolide sesquiterpene lactone [33]. Further evidence to substantiate presence of the epoxide moieties was corroborated by the carbon chemical shift signals appearing at δ_C_ 76.5 (C-1), 58.5 (C-2), 57.0 (C-3), and 70.0 (C-4) (Fig. 3.14), on the ^13^C NMR spectrum which exhibited a downfield shift in a region for epoxide oxygen bearing carbons [34]. Comparison of ^1^H NMR chemical shifts of compound **2** to that of literature showed they were similar to those of the compound reported previously by Koreeda et al. [35], therefore this compound was identified as yomogiartemin.

## Discussion

Extracts of plants are inherently a complex mixture of different compounds. For the purpose of drug discovery these mixtures of compounds need to be separated and the active compounds isolated and subsequently identified. Different approaches, such as ligand fishing-based isolation [36] and the recently reported NMR-based metabolomics-guided approach [37], can be employed. However, in the malaria field the most commonly adopted approach remains classical bioassay-guided isolation [38, 39]. The standard procedure for this approach dictates that only active fractions, as indicated by their activity in biological assays, primarily against intra-erythrocytic asexual *Plasmodium* parasites, are further purified. While bioassay-guided fractionation can be criticised for being time consuming and resource intensive [40], the approach compensates for these shortcomings by its effectiveness. In this study, the bioassay-guided procedure was used to isolate and identify compounds from *A. afra* primarily active in vitro against late-stage gametocytes of *P. falciparum* parasites. This resulted in the discovery and isolation of two previously undocumented gametocytocidal sesquiterpene lactone compounds.

The two compounds, 1α,4α-dihydroxybishopsolicepolide and yomogiartemin, were both found to be the principal components responsible for the gametocytocidal activity of *A. afra*. Both these compounds fall under the guaianolide class of sesquiterpene lactones. Structurally these compounds vary in that 1α,4α-dihydroxybishopsolicepolide is highly oxygenated while yomogiartemin has two epoxide groups. While information on the pharmacological activity of yomogiartemin is relatively scant, 1α,4α-dihydroxybishopsolicepolide has been shown to be active in vitro against chloroquine-sensitive intra-erythrocytic asexual *P. falciparum* (PoW strain) parasites with a reported IC_50_ value of 8.7 µg/ml (27.2 µM) [25] which compares favourably to an IC_50_ value of 6.0 µg/ml (18.7 µM) measured in this study for this compound against asexual chloroquine-sensitive *P. falciparum* (NF54 strain) parasites. The work presented in this study was the first to provide a report on the gametocytocidal activity of these two compounds. Both compounds showed good activity against early- and late-stage gametocytes with IC_50_ values of < 10 µg/ml. Interestingly, 1α,4α-dihydroxybishopsolicepolide was significantly more potent against late-stage gametocytes than early-stage gametocytes and intra-erythrocytic asexual *P. falciparum* parasites. This is consistent with a previous report showing that the crude extract of *A. afra* was more active against late-stage gametocytes than early-stage gametocytes [17]. The isolated compounds were also active against asexual parasites of *Plasmodium*, marking them as potential pan-reactive anti-malarial drug candidates. This pattern of in vitro pharmacological activity has been observed for the sesquiterpene lactone, artemisinin, which is known to exhibit pan-reactivity in vitro [41–43]. However, artemisinin is more potent (double to single digit ng/ml IC_50_ values) across all three stages investigated [27, 42–44] although moderate activity of this compound and its derivatives has been reported against the mature, stage-V gametocytes (reported IC_50_ of > 12.5 µM, > 90% stage V) [13]. Parthenin, a sesquiterpene lactone of the guaianolide class [45], and parthenolide, a member of the germacranolide class and additionally has a single epoxide ring, have both been reported to be highly potent against stage-V gametocytes [22]. Data presented in this study along with that from other investigations [18, 22] strongly point to sesquiterpene lactones being a chemical scaffold worthy of serious consideration in development as target candidate product-5 molecules, that is, drugs prioritized towards transmission-blocking purposes [46].

Sesquiterpene lactones have been known to exert their therapeutic effect through a number of biological activities [47–50]. Mode of action (MOA) of this class of compounds has been attributed to the alkylation of biological molecules by the α-methylene-γ-lactone group. Reported effects include disruption of ion homeostasis state, generation of reactive oxygen species and induction of apoptosis [48, 51]. Despite the known gametocytocidal activity of sesquiterpene lactones, their MOA against malaria parasites, gametocytes in particular, still remains poorly understood [22]. However, recent evidence suggesting that interruption of mature stage-V gametocyte homeostasis is a potential MOA for some gametocytocidal drugs [13], does provide a clue on the potential MOA of sesquiterpene lactones against gametocytes. This may be due to inhibition of the sarco/endoplasmic reticulum Ca^2+^-ATPase (SERCA) pump, which has been shown to be targeted by two other sesquiterpene lactones, thapsigargin and artemisinin [49]. Inhibition of the SERCA pump leads to elevated cytoplasmic levels of calcium leading to disruption of cellular homeostasis balance [47]. Primaquine has been suggested to exert its gametocytocidal activity by production of highly reactive oxygen radicals leading to oxidative stress [52]. Drug-induced oxidative stress could partly account for the gametocytocidal activity of the compounds isolated in this study, particularly yomogiartemin which contains two epoxide rings both of which are capable of producing oxygen radicals.

Unfortunately, the same mechanism in which sesquiterpene lactones exert their therapeutic value may also be responsible for their cytotoxicity [49, 51]. This comes as the Michael-type addition reactions of the α-methylene-γ-lactone are non-specific [51]. It was as such expected that the two isolated compounds were going to be cytotoxic. However, this proved not to be the case as both compounds showed negligible effect on the HepG2 cells at concentration points almost equal to the IC_50_ values (< 10 µg/ml) against the asexual parasites of *Plasmodium*.

Synergy and additive effects in crude extracts of plants have been observed in other investigations, in some cases leading to greater activity in comparison to pure isolated compounds [53]. This prompted the comparison of activity of the chloroform fraction of *A. afra* to that of the two gametocytocidal compounds isolated in this study. The pure compounds were more selective towards the gametocyte stages while activities against asexual stages were identical to that of the chloroform fraction. However, activity against gametocytes was still in the same range as that observed for the crude extract of *A. afra* [17]. Hence, compared to the crude extract of *A. afra*, the activities of the two isolated compounds were not better than the crude extract despite the fact that concentration of the active compounds, on mass basis, was several orders of magnitude lower in the crude extract than in the pure compounds dosed. This pharmacological effect can partly be explained by the possible synergistic role played by other natural product compounds in *A. afra*. These could aid in the uptake of the active compounds, inhibit extrusion pumps, decrease metabolism or transport of the active compound leading to either synergistic or additive effects. Such effects have been observed for whole *A. annua* compared to pure artemisinin, with the former containing same concentration of the latter during the investigation. The whole dry plant was seen to be more active in vivo than the single pure compound, with the authors explaining that this was possibly due to improved bioavailability as well as synergism with other compounds, in particular the flavonoids [54]. The authors explain that flavonoids such as quercetin, which is found in *A. annua*, may possibly potentiate artemisinin activity by inhibiting the enzyme thioredoxin reductase, which plays a vital role in the maintenance of redox balance in the intra-erythrocytic asexual parasites of *Plasmodium* [54] making it more sensitive to drug-induced oxidative stress. Susceptibility of mature stage-V *Plasmodium* gametocytes to disruption in redox balance has been reported recently [55]. The authors demonstrated that compounds which inhibit enzymes critical in maintaining redox balance do potentiate drugs such as methylene blue which act by inducing oxidative stress [55]. *Artemisia afra* is a known producer of flavonoids [25] including luteolin [56] which structurally is similar to quercetin [57]. It is thus possible that these flavonoids may have synergistic effect with the sesquiterpene lactones. Additive activity within sesquiterpene lactones against asexual parasites of *Plasmodium* has been also reported in other studies [58]. There is a need to further explore and investigate synergy and additive activity within sesquiterpene lactone compounds and sesquiterpene lactones combined to flavonoids, against both gametocyte stages and intra-erythrocytic asexual *Plasmodium* parasites.

## Conclusions

Plants remain poorly investigated for their activity against the sexual transmittable gametocyte stages of the malaria parasite. The current study has clearly shown that a bioassay-guided strategy can be adopted fruitfully using phenotypic screening, which led to the isolation of two sesquiterpene compounds: 1α,4α-dihydroxybishopsolicepolide and yomogiartemin. Both of these have novel gametocytocidal activity, with negligible overt cytotoxicity. These compounds are unique starting points for their optimization to develop safe and efficacious pan-reactive transmission-blocking anti-malarial drugs.

## Additional file

**Additional file 1: Table S1.** Inhibition of *in vitro* viability of late stage gametocytes of *Plasmodium falciparum* (NF54 strain) by crude extract and fractions of *Artemisia afra.* **Table S2.** Inhibition of *Plasmodium falciparum* late gametocyte stages by fractions from column 1. **Table S3.** IC_50_ values of *Artemisia afra* chloroform fraction, compounds 1 and 2 on intra-erythrocytic asexuals, early gametocytes and late stage gametocytes. **Fig. S1.** Base Peak Ion chromatograms from UPLC-MS analysis using ESI +ve mode for fractions A) F10, B) F11, C) F13 and D) F19. **Fig. S2.** Full dose-response curve plots for artemisinin (ART) and methylene blue (MB) against late-stage *Plasmodium falciparum* gametocytes.

## Abbreviations

pLDH: parasite lactate dehydrogenase
ACT: artemisinin-based combination therapy
WHO: World Health Organization
NMR: nuclear magnetic resonance
UPLC: ultra-high pressure liquid chromatography
MS: mass spectrometer
DEPT: distortionless enhancement by polarisation transfer
HSQC: heteronuclear single quantum coherence
HMBC: heteronuclear multiple bond correlation
NOESY: nuclear overhauser effect spectroscopy
COSY: correlation spectroscopy
