# Supplementary material for: Bioassay-guided isolation and identification of gametocytocidal compounds from Artemisia afra (Asteraceae)
Source: Malar J. 2019 Mar 8;18:65. doi: 10.1186/s12936-019-2694-1 (PMC6408838; doi:10.1186/s12936-019-2694-1)

**Additional Files**

**Table S1 Inhibition of *in vitro* viability of late stage gametocytes of *Plasmodium falciparum* (NF54 strain) by crude extract and fractions of *Artemisia*** ***afra***

| Fraction of *Artemisia afra* | % Inhibition of viability | |
| --- | --- | --- |
|  | **5 µg/ml** | **10 µg/ml** |
| Crude extract | 8.9±3.0 | 21.1±7.4 |
| Hexane | 9.3±4.3 | 7.0±7.4 |
| Chloroform | 55.8±3.0 | 99.0±0.2 |
| Ethyl acetate | 10.6±4.2 | 19.2±2.4 |
| Methanol | 9.7±6.2 | 3.3±3.4 |

**Table S2 Inhibition of *Plasmodium falciparum* late gametocyte stages by fractions from column 1**

| Fraction | % Inhibition of viability | | Fraction | % Inhibition of viability | |
| --- | --- | --- | --- | --- | --- |
|  | **5 µg/ml** | **10 µg/ml** |  | **5 µg/ml** | **10 µg/ml** |
| F1 | 37.7±8.8 | 60.0±0.8 | F13 | 87.6±1.7 | 100±1.1 |
| F2 | 38.0±8.7 | 75.3±6.7 | F14 | 48.3±3.1 | 97.0±0.8 |
| F3 | 53.5±3.7 | 95.2±2.4 | F15 | 25.0±8.2 | 38.4±3.4 |
| F4 | 45.1±6.0 | 90.8±3.5 | F16 | 45.7±3.0 | 92.3±0.7 |
| F5 | 44.1±3.6 | 77.5±8.3 | F17 | 30.7±5.2 | 74.5±6.3 |
| F6 | 40.4±5.9 | 75.5±10.1 | F18 | 41.7±1.5 | 58.5±3.0 |
| F7 | 45.0±3.6 | 93.8±3.9 | F19 | 47.4±5.5 | 85.6±5.2 |
| F8 | 37.6±5.5 | 93.6±3.6 | F20 | 21.4±3.0 | 55.5±6.0 |
| F9 | 33.6±0.9 | 57.3±0.4 | F21 | 9.0±4.1 | 2.0±3.0 |
| F10 | 78.3±5.6 | 99.2±1.84 | F22 | 25.3±0.9 | 36.1±2.3 |
| F11 | 67.3±7.6 | 99.2±0.6 | F23 | 32.0±6.5 | 52.1±3.9 |
| F12 | 43.5±5.1 | 85.0±1.6 | F24 | 14.2±5.4 | 35.6±0.8 |

**Table S3 IC_50_ values of *Artemisia afra*** **chloroform fraction, compounds 1 and 2 on intra-erythrocytic asexuals, early gametocytes and late stage gametocytes**

|  | IC_50_ value (µg/ml) | | |
| --- | --- | --- | --- |
|  | **Asexual stages** | **Early stage gametocytes** | **Late stage gametocytes** |
| *Artemisia afra* chloroform fraction | 6.3±2.4 | 7.8±0.3 | 5.6±0.2 |
| Compound 1 | 6.0±0.6 (18.7 µM) | 5.6±0.2 (17.5 µM) | 2.0±0.2 (6.3 µM) |
| Compound 2 | 3.8±1.2 (11.3 µM) | 4.4±0.3 (13.1 µM) | 5.3±0.5 (15.8 µM) |

**Fig. S1 Base Peak Ion chromatograms from UPLC-MS analysis using ESI +ve mode for fractions** **A) F10, B) F11, C) F13 and D) F19**

In addition to showing high activity against late-stage gametocytes of *P. falciparum*, F13 and F19 were finally selected ahead of F10 and F11, as both the former-mentioned fractions had fewer compounds, which was deemed an advantage as it meant fewer challenges to be encountered in further chromatographic purification.


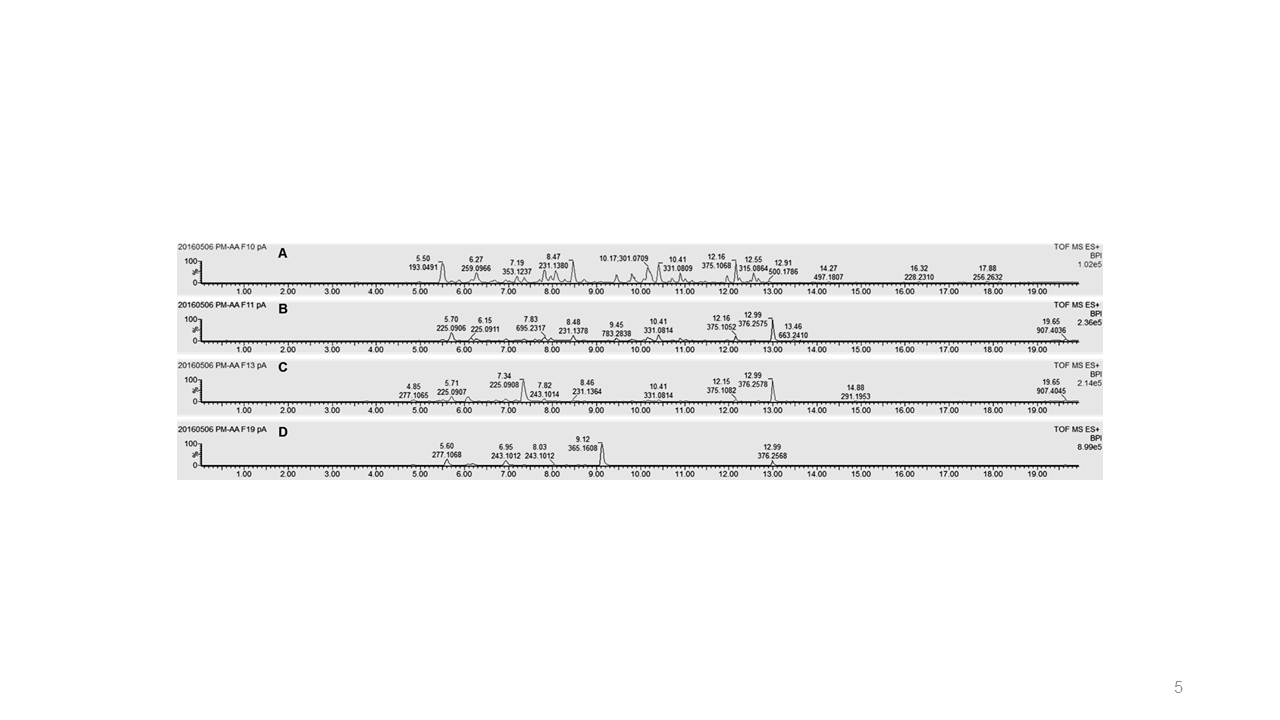


**Fig. S2 Full dose-response curve plots for artemisinin (ART) and methylene blue (MB) against late-stage *Plasmodium falciparum* gametocytes**

IC_50_ values determined using the 72+72 h pLDH assay using 10-fold serial dilution (*n*=1, carried out in technical triplicates).


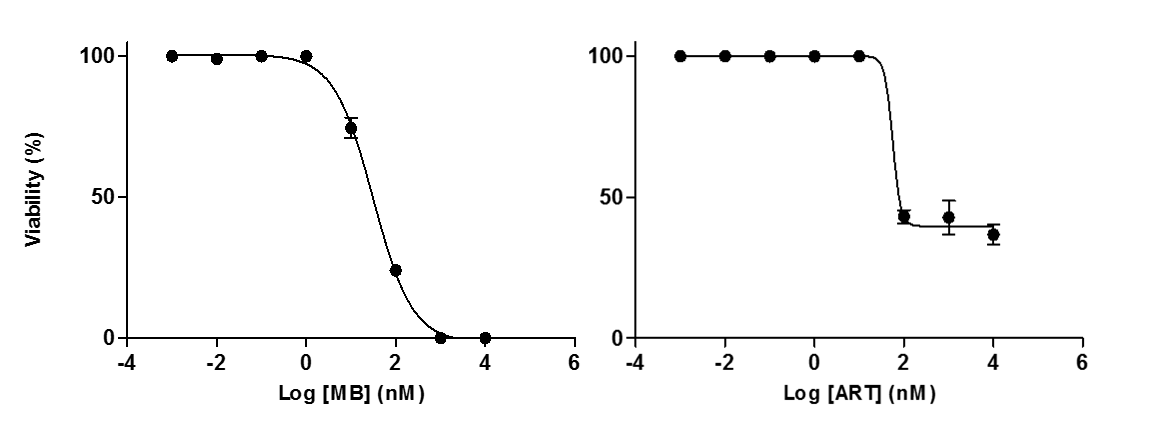

Supplement: Supplementary file 1 — Additional file 1: Table S1. Inhibition of in vitro viability of late stage gametocytes of Plasmodium falciparum (NF54 strain) by crude extract and fractions of Artemisia afra. Table S2. Inhibition of Plasmodium falciparum late gametocyte stages by fractions from column 1. Table S3. IC50 values of Artemisia afra chloroform fraction, compounds 1 and 2 on intra-erythrocytic asexuals, early gametocytes and late stage gametocytes. Fig. S1. Base Peak Ion chromatograms from UPLC-MS analysis using ESI +ve mode for fractions A) F10, B) F11, C) F13 and D) F19. Fig. S2. Full dose-response curve plots for artemisinin (ART) and methylene blue (MB) against late-stage Plasmodium falciparum gametocytes. [file 12936_2019_2694_MOESM1_ESM.docx]
